# Resilience in Bipolar Disorder Compared to Clinical and Non‐Clinical Populations: A Systematic Review and Meta‐Analysis

**DOI:** 10.1111/acps.70042

**Published:** 2025-11-12

**Authors:** Derek Clougher, Michele De Prisco, Brisa Solé, Laura Montejo, Maria Serra‐Navarro, Maria Florencia Forte, Patricia Camprodon‐Boadas, Elena de la Serna, Vincenzo Oliva, Sara Martin‐Parra, Jose Sánchez‐Moreno, Benedikt L. Amann, Marina Garriga, Norma Verdolini, Marta Ribases, Kamilla Miskowiak, Anabel Martínez‐Aran, Eduard Vieta, Silvia Amoretti, Carla Torrent

**Affiliations:** ^1^ Departament de Medicina, Facultat de Medicina i Ciències de la Salut, Institut de Neurociències (UBNeuro) Universitat de Barcelona (UB) Barcelona Spain; ^2^ Bipolar and Depressive Disorders Unit, Hospital Clínic de Barcelona Barcelona Spain; ^3^ Fundació Clínic‐Institut d’Investigacions Biomèdiques August Pi I Sunyer (IDIBAPS) Barcelona Spain; ^4^ Centro de Investigación Biomédica en Red de Salud Mental (CIBERSAM) Instituto de Salud Carlos III Barcelona Spain; ^5^ BIOARABA, Department Psychiatry, Hospital Universitario de Alava University of the Basque Country Vitoria Spain; ^6^ Department of Child and Adolescent Psychiatry and Psychology, 2021SGR01319 Hospital Clínic de Barcelona Barcelona Spain; ^7^ Department of Psychiatry, Hospital del Mar, Hospital del Mar Medical Research Institute University Pompeu Fabra Barcelona Spain; ^8^ Local Health Unit Umbria 1, Department of Mental Health, Mental Health Center of Perugia Perugia Italy; ^9^ Group of Psychiatry, Mental Health and Addictions, Vall d’Hebron Research Institute (VHIR), Vall d’Hebron Research Institute (VHIR) Barcelona Catalonia Spain; ^10^ Neurocognition and Emotion Across Disorders of the Brain Centre (NEAD), Copenhagen Mental Health Centre, Frederiksberg Hospital Frederiksberg Denmark

**Keywords:** bipolar disorder, meta‐analysis, resilience, severe mental illness, systematic review

## Abstract

**Introduction:**

Resilience is present in both clinical and non‐clinical populations; yet, there is a paucity of literature examining its role in bipolar disorder (BD). The goal of the present systematic review and meta‐analysis was to substantiate the extant literature investigating resilience in BD in comparison to clinical and non‐clinical populations.

**Method:**

PubMed/MEDLINE, PsycINFO, and Scopus were systematically searched from inception to August 8th, 2024.

**Results:**

Twenty‐eight studies using a validated resilience scale with a total of 3094 people with BD, 4100 healthy controls, and 1768 with other mental diagnoses were included in the systematic review, and 21 were analyzed in a random effects meta‐analysis. A statistically significant result with a medium effect (SMD = −0.787, *p* < 0.001) indicated that people with BD reported lower levels of resilience than healthy controls. Similarly, patients with BD showed higher levels of resilience than patients with schizophrenia (SCZ) (SMD = 0.336, *p* = 0.013). No significant differences were found between BD and major depressive disorder (MDD).

**Conclusion:**

Findings should be interpreted with caution due to the high heterogeneity observed and methodological challenges in the definition and measurement of resilience. Future research should aim to better characterize resilience in BD by improving its assessment as a standardized element of clinical evaluation. This will provide a basis for strategies to reduce the burden of this chronic condition.

**Summary**

- Summations
  ○ Bipolar disorder reported lower resilience than controls and greater resilience than schizophrenia.
  ○ No significant differences in resilience were found between bipolar disorder and major depressive disorder.
  ○ Euthymia was associated with less differences in resilience between bipolar disorder and controls.
- Limitations
  ○ Only two studies included were longitudinal, limiting understanding of resilience in bipolar disorder over time.
  ○ All included studies used self‐report measures of resilience, highlighting a need for more objective, ecologically valid measurement tools for resilience.
  ○ The small sample size may limit robustness of findings.

## 1 Introduction

Bipolar disorder (BD) is a chronic mental illness characterized by recurrent mood variations including manic, hypomanic, and depressive episodes alongside intermittent periods of euthymia or subthreshold symptomatology [1]. BD is the sixth leading cause of global disability [2]. Early onset in BD is common with a mean onset age of ~20 years, and delays in diagnosis of up to 10 years, preventing adequate treatment despite evidence that early intervention and diagnosis are key in promoting recovery [1, 3]. BD is not confined to mental health difficulties; patients also report lower quality of life in terms of physical health [4, 5] and have a reduced life expectancy in comparison to the general population [6].

The impact of BD consists of clinical symptomatology as well as functional and cognitive impairment [7, 8]. Functional impairment has been reported both in premorbid and early stages and during remission, affecting multiple domains including autonomy, occupational and educational achievement, cognition, and interpersonal relationships [9], exacerbating the condition [10]. Functional recovery remains a core challenge in BD as patients show higher rates of clinical remission; 64% of patients in outpatient care achieved clinical remission 2 years post‐treatment in comparison to only 34% functional remission in Haro et al. [11]. Similarly, cognitive impairment is evident across multiple domains and illness stages [7, 12], and has been linked to poorer functional outcomes [13]. Approximately 50% of patients experience persistent cognitive impairment, yet no evidence‐based pharmacological treatments are currently available to address it [14]. Furthermore, although various interventions aim to improve overall clinical, functional, and cognitive symptoms of BD, there is no complete cure for the disorder [15]. Consequently, it is imperative that people with BD are offered the opportunities not just to do better, but also to feel and live better in the context of this chronic condition [16].

A paradigm shift towards mental health promotion is increasingly seen as an approach to improve overall well‐being, helping people live better with their illness [17]. A core aspect of well‐being is resilience, which is an important aspect of both prevention and intervention in mental illness [18]. Although a consensus regarding the definition of resilience has not yet been reached, the APA Dictionary cites resilience as the “process and outcome of successfully adapting to difficult or challenging life experiences, especially through mental, emotional, and behavioural flexibility and adjustment to external and internal demands” [19]. Furthermore, the APA also includes in their definition that resilience is “the process of adapting well in the face of adversity, trauma, tragedy, threats, or significant sources of stress‐such as family and relationship problems, serious health problems, or workplace and financial stressors” which also includes “bouncing back…personal growth…[and] coping strategies” [20]. Importantly, resilience is considered a dynamic process influenced by interactions among many factors including environment, social support systems, and individual development while also highlighting that there are inconsistencies in how researchers define resilience [21]. Resilience has been frequently, and controversially, cited as the absence of mental illness or the maintenance of mental health when an individual experiences trauma and/or adversity [22]. This narrow perspective is problematic, as detailed by a recent multidisciplinary expert panel on resilience (see Denckla et al. [20]) as: (i) a dichotomic approach fails to account for the periods of instability characteristic in severe mental illness (SMI); (ii) it does not consider an individual's mental health prior to the adversity; (iii) assigning appropriate control groups is challenging as it requires a control group that has never faced adversity; (iv) it adopts a one‐size‐fits‐all approach across disorders and does not address specific diagnostic criteria. Regarding the latter, this is problematic because post‐traumatic stress disorder (PTSD) is only diagnosed when trauma is present, whereas BD does not require this criterion to receive an official diagnosis [19]. Furthermore, effectively measuring resilience remains complex; there is an over‐reliance on self‐report measures, which, in the context of SMI, may be impacted by illness stage, current mood symptoms, as well as cognitive and functional difficulties.

An additional challenge to understanding resilience in BD is the fact that a wealth of research has focused solely on the study of resilience in PTSD and major depressive disorder (MDD), thus providing limited insights into resilience in other mental disorders [23]. Greater resilience has been associated with less severe symptoms [24] and better treatment outcomes in PTSD [25]. Moreover, people diagnosed with MDD report lower levels of resilience than healthy controls (HCs) [24], which is also linked to poorer treatment response [26, 27]. Importantly, resilience appears to represent a potential protective factor in SMI as it moderated the risk of depression in suicidal patients [28] and was also associated with reduced suicidal ideation [29]. Furthermore, lower resilience levels have been reported in individuals with schizophrenia (SCZ) and at clinical high risk for psychosis [30]. Higher resilience was associated with reduced negative symptoms, depression, and anxiety in individuals at clinical high risk for psychosis [31]. However, a deeper understanding of resilience in BD and its potential protective properties is needed to identify ways to incorporate its assessment and strategies to target resilience in clinical practice.

### 1.1 Rationale and Aim

There is a paucity of research exploring resilience in BD. To our knowledge, to date only two reviews investigating resilience in BD have been conducted [32, 33], and certain limitations must be considered. Chan et al. [31] systematically reviewed the evidence for resilience in BD. Imrana et al. [29] adopted a meta‐analytic approach but were not specifically focused on BD. Thus, despite their contribution, these reviews offer limited insight in drawing substantial conclusions beyond the scope of their individual objectives. Consequently, a systematic review and meta‐analysis with a specific focus on BD using stricter criteria is warranted. Accordingly, the aim of the current paper was to examine resilience in BD vs. clinical and non‐clinical populations via a systematic review and meta‐analysis and identify differences in total resilience scores and sub‐domains.

## 2 Materials and Methods

The Preferred Reporting Items for Systematic Reviews and Meta‐Analyses (PRISMA) guidelines [34] were used to conduct this systematic review and meta‐analysis. The PRISMA checklist is reported in Supporting Information: Appendix I. The protocol and search strategy for this study were registered on PROSPERO (CRD42023440501). Deviations from the original protocol are reported in Supporting Information: Appendix II.

### 2.1 Eligibility Criteria

Eligibility criteria were followed using the Population, Intervention, Comparison, Outcome (PICO) framework. The inclusion criteria were: (1) Population: individuals diagnosed with BD according to diagnostic criteria from the Diagnostic and Statistical Manual for Mental Disorders (DSM) (APA, 1994, 2000, 2013) or the International Classification of Diseases (ICD) (WHO, 2004); (2) Intervention: not applicable; (3) Comparison: any study comparing people with BD to any other clinical (people affected by any other psychiatric condition, officially diagnosed following clinical guidelines) or non‐clinical (healthy controls [HCs], unaffected first‐degree relatives) population; and (4) Outcome: original studies with quantitative data for resilience as measured via a psychometrically validated measurement tool for resilience (any measurement tool that has been previously tested for reliability and validity in peer‐reviewed research).

No restrictions for sample size, age, or language were applied. Both observational (cross‐sectional and longitudinal) and intervention studies were eligible for inclusion. For the longitudinal and intervention studies, only baseline data was considered. In studies using overlapping populations, we chose the study with the most complete dataset relevant to our objectives. Exclusion criteria were: (1) reviews, meta‐analyses, case reports, case studies, and animal studies given their lack of control group and/or incompatibility with our research aims; (2) non‐peer‐reviewed literature such as books, book chapters, commentary pieces, PhD theses, and conference posters; (3) studies using non‐validated measures of resilience; and (4) any study that does not meet inclusion criteria.

### 2.2 Search Strategy

The PubMed/MEDLINE, PsycINFO, Scopus, and PsycInfo databases were systematically searched from inception until August 8th, 2024. Search strings are available in Supporting Information: Appendix III. A backward snowballing technique, a method to identify relevant research by examining reference lists, was used to review the sources found within the reference lists of the identified literature to search for any additional papers not found in the original search.

### 2.3 Procedure and Data Extraction

Retrieved articles were imported into Mendeley (https://www.mendeley.com) and duplicate articles were removed. All studies were independently screened by title and abstract using Rayyan by five authors (S.A., D.C., L.M., B.S., C.T.). When a consensus was not reached, another author (M.D.P.) was consulted. The remaining articles were independently reviewed by two authors (S.A., D.C.) at the full‐text level, and M.D.P. was consulted when a consensus was not reached.

Two authors (S.A., D.C.) independently extracted data from the articles included. Data extraction included, when available: first author, publication year, geographical region/country, study design, method to establish diagnostic criteria, (semi)structured interview administered, study setting, resilience scale used, resilience domain, control group, total number of cases and controls, mean and standard deviation (SD) of outcomes for cases and controls, mean age of cases and controls, % of females for cases and controls, age at onset of BD, duration of illness (DOI), % of BD‐I among cases, % of people in euthymia, depression, or (hypo)mania among cases, % of patients prescribed psychotropic medication, psychiatric and/or medical comorbidities in cases and clinical controls, and depressive or manic symptom severity scale mean scores for cases and controls. For studies solely using data reported in figures, we used WebPlotDigitizer to conduct manual extraction. When required information was unavailable in the paper, the corresponding authors were contacted twice to request the data.

### 2.4 Quality Appraisal

Two authors (S.A., D.C.) independently assessed the risk of bias using the Newcastle‐Ottawa Scale (NOS) [35], and disagreements were resolved by another author (C.T.). The NOS contains three sections evaluating the following three quality parameters: selection, comparability, and outcome. Each parameter is evaluated across seven items with scores ranging from 0 to 10 points. Scores are used to rate the study from unsatisfactory to very good. The NOS scores were converted to Agency for Healthcare Research and Quality (AHRQ) standards as described in Oliva et al. [36] and the scoring guidelines are as follows: 5–6 “satisfactory”, 7–8 “good”, and 9–10 “very good”.

### 2.5 Statistical Analyses

Statistical analyses were performed using RStudio R version 4.3.1 (R Core Team, 2022), and the meta‐analysis was performed via the metafor package for R [37] using a random‐effect model (restricted maximum‐likelihood estimator) [38]. Standardised mean differences (SMD) with 95% confidence intervals (CI) represented by Hedge's g were used to calculate effect sizes. The following interpretation of SMD values can be made: 0.2–0.5 (small), 0.5–0.8 (medium), < 0.8 (large). An SMD of 0 means that there is no difference between groups, and an SMD that is negative suggests that the experimental group has a lower mean score than the control group [39]. 95% prediction intervals (PIs) were also calculated to indicate the range in which the true effect of a future similar study is expected to fall, accounting for between‐study variability. A leave‐one‐out sensitivity analysis, which consists of removing one study at a time from the analysis, was performed to assess the result's robustness. Cochran's Q [40], τ2 and I2 were used to assess the heterogeneity. Prediction intervals were also estimated [41]. In results of *p* < 0.1 in Cochran's Q and an I2 with a value of > 50%, meta‐regression analyses of subgroups pre‐specified in the study protocol, that is, mean age of the cases, age at onset of BD, DOI, % of females among the cases, % of people with BD‐I, % of people in euthymia, and depressive and manic symptoms severity scale mean scores, were performed. Publication bias was examined via funnel plots and using the Egger's test [42] when at least 10 studies were available.

## 3 Results

**FIGURE 1:**
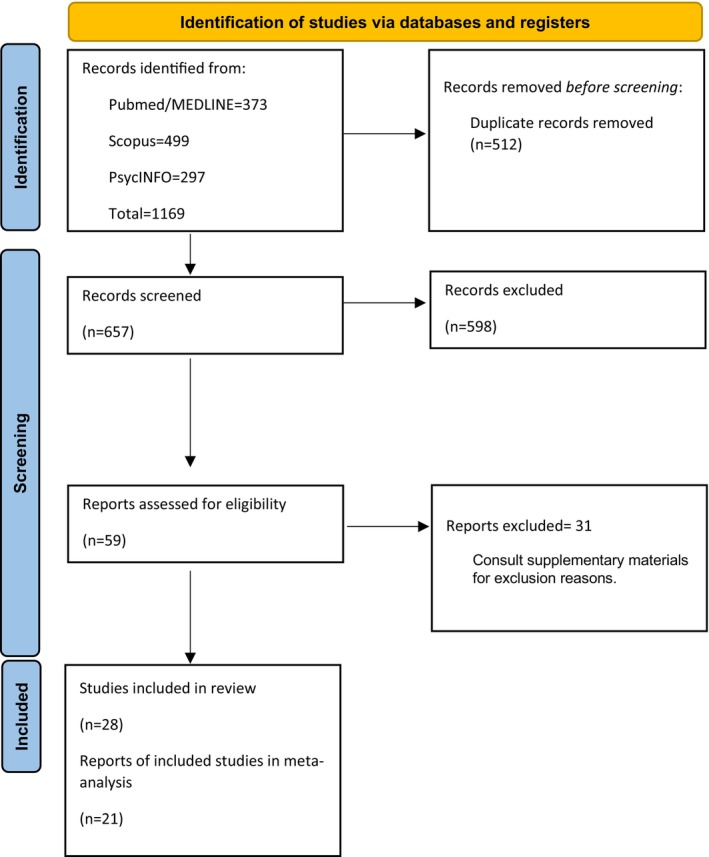
PRISMA flowchart of overall study selection process.

The overall study selection process is shown in the PRISMA flowchart in Figure 1. One thousand one hundred sixty‐nine articles were identified in the database search. Five hundred twelve duplicates were identified in Rayyan and removed. Six hundred fifty‐seven articles underwent screening, and 598 articles were removed at the first stage title and abstract level. Fifty nine articles underwent full‐text evaluation, and a total of 31 were excluded. 28 studies were included in this systematic review, with 21 [43, 44, 45, 46, 47, 48, 49, 50, 51, 52, 53, 54, 55, 56, 57, 58, 59, 60, 61, 62, 63] providing sufficient data to perform the meta‐analysis. Authors reporting on resilience and BD compared to other clinical/non‐clinical populations were contacted twice to request the statistical analysis when data were not provided. Ten authors were contacted, and two replied with the requested analysis, making them eligible for inclusion [60, 63]. Excluded articles with reasons are provided in Supporting Information: Appendix IV.

### 3.1 Study Characteristics

Included studies' characteristics are provided in Table 1. All 28 papers were published between 2015 and 2024. The total patient sample across studies was 4862 participants. Three thousand ninety‐four patients were diagnosed with BD, 1432 with MDD, and 336 with SCZ. The total population of HCs was 4100. All studies included adults (age range: 22–77) using cross‐sectional data [42, 43, 44, 45, 46, 47, 49, 50, 51, 52, 53, 54, 55, 56, 57, 58, 59, 61, 62, 63, 64, 65, 66, 67, 68, 69] except for two studies with longitudinal data [60, 70]. Only baseline data was used for these studies.

**TABLE 1:** Study characteristics.

Authors and year	Country	Study design	Setting	Population *n*	Mood state (%)	Sex—female *N* (%)	Primary study aim	Resilience measure	Diagnostic scale(s)	Study quality (NOS)
Aslan and Baldwin 2021	Turkey	Cross‐sectional	Outpatients	50 BD, 50 MDD, 50 HCs	Depressed (100)	105 (70)	Examine differences MDD/BD/HCs in rumination, cognitive functions, emotion regulation, psychological resilience.	BRS	DSM‐5 SCID	Very good
Bozikas et al. 2018	Greece	Cross‐sectional	Outpatients	40 BD, 40 HCs	N/A	164 (85.3)	Explore relationship between resilience and social functioning.	CD‐RISC	DSM‐IV MINI	Good
Chiang et al. 2024	USA	Longitudinal	Outpatients	100 BD	Depressed (80) Manic (11) Hypomanic (7) Euthymia (2)	58 (58)	Explore the potential effect of resilience in enhancing treatment response at different speciality treatment sessions.	RBD	DSM‐5 SCID	Satisfactory
Choi et al. 2015	Republic of Korea	Cross‐sectional	Outpatients	62 BD, 62 HCs	Euthymia (100)	70 (56.4)	Investigate the demographic and clinical factors related to resilience in euthymic patients with BD.	CD‐RISC	DSM‐IV‐TR	Good
Chung et al. 2018	Republic of Korea	Cross‐sectional	Outpatients	77 BD, 224 MDD, 958 HCs	N/A	745 (59.1)	Investigate the relationship between morningness‐eveningness and resilience in patients with a mood disorder.	CD‐RISC	DSM‐IV	Good
Craba et al. 2023	Italy	Cross‐sectional	Outpatients	55 BD, 51 MDD, 60 HCs	N/A	102 (61.4)	Analyze resilience and attachment styles in patients with MDD, BD and HCs.	CD‐RISC	DSM‐V‐TR	Good
Datta and Chetia 2023	India	Cross‐sectional	Inpatients and Outpatients	30 BD, 30 SCZ	N/A	21 (35)	Assess and compare the influence of resilience and internalized stigma on treatment effectiveness in patients with BD and SCZ in a tertiary care facility	CD‐RISC	ICD‐10	Good
Deng et al. 2018	China	Cross‐sectional	Outpatients	34 BD 81 SCZ 52 HCs	N/A	76 (46)	Compare resilience and cognitive function in patients diagnosed with SCZ, BD, and HCs.	CD‐RISC	DSM‐IV	Good
Dou et al. 2022	China	Cross‐sectional	Inpatients and Outpatients	246 BD, 69 HCs	N/A	198 (62.8)	Explore mediating effects of social support, resilience and suicidal ideation on the relationship between family and psychosocial functioning in BD.	CD‐RISC	DSM‐V Axis I Disorders (SCID‐CV)	Good
Echezarraga et al. 2017	Spain	Longitudinal	Outpatients	115 BD, 71 HCS	N/A		Develop a measurement tool for resilience in BD.	RBD	DSM‐IV	Very good
Favale et al. 2023	Italy	Cross‐sectional	Outpatients	38 BD, 31 MDD	N/A	38 (55.1)	Explore associations between hope, resilience, socio‐demographic and clinical characteristics in BD vs. MDD	CD‐RISC	DSM‐5	Good
Hofer et al. 2017	Austria	Cross‐sectional	Outpatients	60 BD, 52 SCZ, 77 HCs	N/A	108 (57)	Investigate health‐related quality of life in severe mental illness and the influence of symptomatic remission and resilience.	RS‐25	DSM‐IV	Good
Just et al. 2022	Germany	Cross‐sectional	Inpatient	6 BD, 13 MDD	N/A	**	Explore loneliness levels in psychiatric vs. Somatic inpatients. Resilience included as predictor variable.	RS‐11	ICD‐10	Good
Kang et al. 2024	South Korea	Cross‐sectional	Outpatient	510 BD, 233 MDD, 818 HCs	N/A	N/A	Explore the mediating effects of resilience and anxiety on morningness‐eveningness and depression.	CD‐RISC	DSM‐IV	Satisfactory
Kesebir et al. 2015	Turkey	Cross‐sectional	Outpatient	100 BD	Euthymia (100)	54 (54)	Investigate effects of childhood trauma and affective temperament on resilience in BD.	RSA	DSM‐IV	Satisfactory
Lee et al. 2017	Republic of Korea	Cross‐sectional	Outpatient	68 BD, 68 HCs	N/A	30 (22)	Investigate the association between resilience in quality of life in patients with BD vs. HCs.	CD‐RISC	DSM‐IVTR	Good
Mackali et al. 2023	Turkey	Cross‐sectional	Outpatient	132 BD	Euthymia (100)	79 (59.8)	Explore the relationships between internalised stigma, self‐compassion and resilience in BD.	RSA	DSM‐V	Very good
Mizuno et al. 2016	Japan	Cross‐sectional	Outpatients	60 BD, SCZ 60, HCs 60	N/A	100 (56)	Identify key correlates of resilience in patients with SCZ and BD.	RS‐25	DSM‐IV	Very good
Montejo et al. 2024	Spain	Cross‐sectional	Outpatients	33 BD	Euthymia (100)	19 (57.6)	Measure and characterize resilience in older age BD and it is associated factors.	CD‐RISC‐10	DSM‐V SCID	Satisfactory
Nunes and da Rocha 2022	Brazil	Cross‐sectional	Inpatients	71 BD, 200 MDD, 113 SCZ	N/A	213 (82.1)	Evaluate resilience in patients with BD, MDD, SCZ and correlate it with clinical measures and quality of life.	RS‐25	N/A	Satisfactory
Palagini et al. 2022	Italy	Cross‐sectional	Inpatients	197 BD	Depressed (48.7) Manic (51.3)	84 (42.6)	Explore relationships among resilience, circadian rhythm disturbances and emotion dysregulation in BD and their association with mood features and suicidal risk.	RSA	DSM‐5	Satisfactory
Park et al. 2023	Republic of Korea	Cross‐sectional	Outpatients	540 BD, 247 MDD, 734 HCs	N/A	951 (63)	Compare and contrast the association between childhood trauma and resilience among individuals with BD, MDD and HCs.	CD‐RISC		Good
Post et al. 2018	Austria	Cross‐sectional	Outpatients	60 BD, 77 HCs	Euthymia (100)	35 (58)	Investigate the impact of resilience, internalized stigma, and residual symptoms on quality of life in clinically stable outpatients with BD‐1.	RS‐25	DSM‐IV	Very good
Sato et al. 2023	Japan	Cross‐sectional	Inpatients and Outpatients	24 BD, 24 HCs	N/A	36 (75)	Explore the relationship between cognitive reserve, resilience and cognition in patients with BD.	RS‐25	DSM‐5	Very good
Şenormancı et al. 2020	Turkey	Cross‐sectional	Outpatients	142 BD	Euthymia (100)	70 (49.3)	Investigate correlations between insight and resilience and their relationship with impulsivity, aggression, alcohol use and affective temperament in BD.	RSA	DSM‐5	Satisfactory
Tsigkaropoulou et al. 2023	Greece	Cross‐sectional	Outpatients	64 BD, 66 MDD, 13 HCs	Euthymia (100)	129 (53)	Explore temperament and character personality dimensions impact self‐reported resilience in BD and MDD.	CD‐RISC	DSM‐5	Good
Uygun et al. 2020	Turkey	Cross‐sectional	Outpatients	90 BD, 30 HCs	Euthymia (100)	81 (67.5)	Investigate the relationship between perceived social support and resilience in BD and HCs.	RSA	SCID	Good
Vieira et al. 2020	Brazil	Cross‐sectional	Outpatients	90 BD, 317 MDD, 837 HCs	N/A	721 (58)	Determine the mediating effect of resilience on the relationship between childhood trauma, BD and MDD.	RS‐25	DSM	Very good

*Note*: BD—Bipolar Disorder; BRS—Brief Resilience Scale; CD‐RISC—Connor‐Davidson Resilience Scale; HCs—Healthy Controls; MDD—Major Depressive Disorder; RBD—Resilience Questionnaire for Bipolar Disorder; RSA—Resilience Scale for Adults; RS—Resilience Scale; SCID—Structured Clinical Interview for DSM; SCZ—Schizophrenia.

60.9% of the BD sample was female. The mean age was 38.88 (SD = 11.46) with an onset age of 25.68 (SD = 9.92) years. The mean duration of illness (DOI) was 11.91 (SD = 10.12). Thirteen studies reported BD type, with 58.92% diagnosed with BD‐I. Eight studies reported mood state, with 57.14% of euthymic participants, 31.59% in a depressive state, and 15.6% in a manic state.

Among the clinical sample, 72.93% of the MDD sample were female. The mean age was 39.90 (SD = 10.62) with an onset age of 39.14 (SD = 14.16) years. The mean DOI was 12.02 (SD = 19.48). Finally, 40.76% of the SCZ sample were female. The mean age was 36.49 (SD = 9.78) with an onset age of 24.3 (SD = 11) years. The mean DOI was 19.40 (SD = 21.20). In HCs, 52.87% were female, with a mean age of 31.15 (SD = 7.33) years.

The quality of the included studies was very good for 7 studies (25%), good for 14 studies (50%), and satisfactory for 7 studies (25%) (see Table 1). Each general score can be consulted in Supporting Information: Appendix V.

### 3.2 Measures Used

Thirteen studies used a version of the Connor‐Davidson Resilience Scale (CD‐RISC) [71] to measure resilience. Twelve studies used the original CD‐RISC scale consisting of 25 items [44, 47, 50, 51, 52, 54, 55, 57, 58, 59, 61, 65], and one study [69] used the shortened CD‐RISC‐10 item version. Seven administered a version of the Resilience Scale [72]. Six studies used the 25‐item version [45, 46, 48, 49, 56, 62], and one [63] used the German short form containing 11 items. A further five studies [53, 66, 67, 68, 73] used the Resilience Scale for Adults (RSA) [74]. One study [43] used the Brief Resilience Scale (BRS) [75], and two [60, 70] used the Resilience Questionnaire for Bipolar Disorder (RBD) [60]. All resilience measures can be consulted in Table 1.

### 3.3 Main Analyses

**FIGURE 2:**
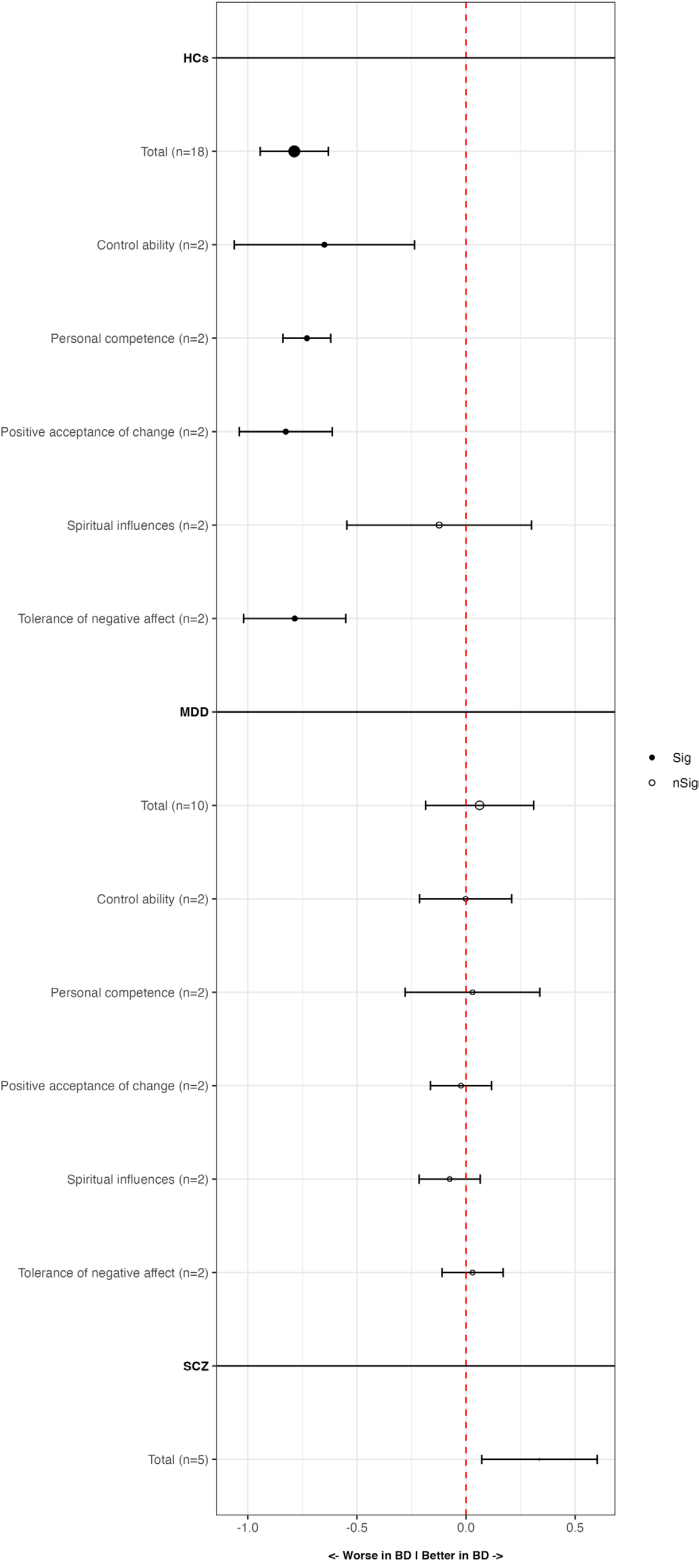
Main results of meta‐analyses.

The main results of the meta‐analyses are presented in Table 2 and Figure 2. The following core analyses were conducted: BD and HCs, BD and MDD, and BD and SCZ.

**TABLE 2:** Results of the meta‐analyses in detail.

Control group	Outcome type	Studies, *n*	BD patients, *n*	Controls, *n*	SMD	95% CIs	*p*	95% PIs	I2	tau2	*Q* test *p* value
HCs	Total	18	2245	4221	**−0.787**	**−0.943, −0.631**	**< 0.001**	**−1.376, −0.198**	82.63	0.08	< 0.1
HCs	Control ability	2	595	794	**−0.649**	**−1.062, −0.236**	**0.002**	−1.317, 0.02	78.68	0.07	< 0.1
HCs	Personal competence	2	595	794	**−0.729**	**−0.839, −0.62**	**< 0.001**	**−0.839, −0.62**	0	0	0.87
HCs	Positive acceptance of change	2	595	794	**−0.826**	**−1.039, −0.613**	**< 0.001**	**−1.127, −0.524**	35.66	0.01	0.21
HCs	Spiritual influences	2	595	794	−0.123	−0.546, 0.3	0.57	−0.811, 0.565	80.07	0.08	< 0.1
HCs	Tolerance of negative affect	2	595	794	**−0.785**	**−1.019, −0.551**	**< 0.001**	**−1.127, −0.443**	44.7	0.02	0.18
MDD	Total	10	1501	1432	0.062	−0.185, 0.31	0.62	−0.676, 0.801	87.46	0.13	< 0.1
MDD	Control ability	2	595	298	−0.002	−0.213, 0.209	0.98	−0.287, 0.283	30.21	0.01	0.23
MDD	Personal competence	2	595	298	0.03	−0.279, 0.338	0.85	−0.438, 0.497	59.42	0.03	0.12
MDD	Positive acceptance of change	2	595	298	−0.023	−0.163, 0.117	0.74	−0.163, 0.117	0	0	0.66
MDD	Spiritual influences	2	595	298	−0.075	−0.215, 0.065	0.29	−0.215, 0.065	0	0	0.5
MDD	Tolerance of negative affect	2	595	298	0.03	−0.11, 0.17	0.67	−0.11, 0.17	0	0	0.45
SCZ	Total	5	255	336	**0.336**	**0.072, 0.601**	**0.013**	−0.184, 0.857	58.28	0.05	< 0.1

*Note*: 95% prediction intervals (PIs) were also calculated to indicate the range in which the true effect of a future similar study is expected to fall, accounting for between‐study variability. SMD values: 0.2–0.5 (small), 0.5–0.0.8 (medium), < 0.8 (large). An SMD of 0 means that there is no difference between groups, and SMD that is negative suggests that the experimental group has a lower mean score than the control group. Significant differences (*p* < 0.05) are marked in bold.

Abbreviations: HCs—healthy controls; MDD—major depressive disorder; SCZ—schizophrenia.

Patients with BD reported significantly lower total resilience scores than HCs (SMD = −0.787; 95% CI = −0.943, −0.631; *p* < 0.001; *I* ^2^ = 82.63). Only two studies [50, 51] provided information regarding differences in subscales of resilience (control ability, personal competence, positive acceptance of change, spiritual influences, tolerance of negative affect) as measured by the CD‐RISC. People with BD showed lower scores in four subdomains (control ability, personal competence, positive acceptance of change, tolerance of negative effect). Similarly, differences in total resilience scores were analyzed in five studies that included a sample of BD and SCZ participants. Significant differences were found as patients with BD reported higher total resilience scores than SCZ (SMD = 0.336; 95% CI = 0.072, 0.601; *p* = 0.013; *I* ^2^ = 58.28). No analysis of subscales was provided in the five included studies that compared BD with SCZ.

No significant differences were found between BD and MDD (SMD = 0.062; 95% CI = −0.185, 0.31; *p* = 0.62; *I* ^2^ = 87.46). Moreover, no significant differences were found in the two studies [50, 51] that included resilience subscales in this comparison.

### 3.4 Meta‐Regression Analyses

Meta‐regression analyses were conducted to explore dichotomic and continuous variables on differences in total resilience scores. When comparing BD and HCs, euthymia in BD was associated with less differences in total resilience scores between both groups (β = 0.07; 95% CI = 0.001, 0.014, *p* = 0.022). In the comparison of BD and MDD, BD‐I diagnosis was associated with greater differences in total resilience scores between BD and MDD (β = 0.009; 95% CI = 0.003, 0.015; *p* = 0.002). No significant differences were found in the meta‐regressions comparing BD and SCZ. No other variables were found to significantly predict differences in total resilience scores. Complete information regarding the meta‐regressions can be consulted in Supporting Information: Appendix VI.

### 3.5 Sensitivity Analysis

Sensitivity analyses were conducted (i) removing one study at a time (leave one out method), (ii) considering good‐quality studies only. Results did not show a change in overall significance level in BD and HCS or in the analysis of BD and MDD. However, in the comparison of BD and SCZ, by removing Deng et al. [52], Mizuno et al. [53], and Nunes and da Rocha [59] the result became non‐significant. Complete information regarding the sensitivity analysis can be found in Supporting Information: Appendix VI.

### 3.6 Publication Bias

The Egger test [42] was performed to investigate publication bias when at least 10 studies were available. Examining the differences in total resilience scores between people with BD and HCs, no publication bias was found (*z* = −1.78; *p* = 0.07). Similarly, the results of the Egger test comparing total resilience scores between people with BD and MDD were also non‐significant (*z* = −0.799; *p* = 0.42). Details on publication bias can be consulted in Supporting Information: Appendix VII.

## 4 Discussion

This systematic review and meta‐analysis examined resilience in BD in comparison with clinical and non‐clinical populations. Two core findings were observed. First, patients with BD report lower levels of resilience in comparison to HCs. Second, patients with BD report higher levels of resilience than patients with SCZ but no significant differences in comparison with MDD. Additionally, meta‐regressions including the period of euthymia and BD‐I provided significant results, offering potential clinical insights suggesting that resilience could fluctuate according to the course of the disorder as well as bipolar type. Overall, the results may guide the research agenda in addressing the limitations in the study of resilience in BD, both in definition and operationalisation, as well as in SMI.

### 4.1 Resilience in BD and Non‐Clinical Populations

In the BD and HC comparison, significant differences were found, with BD reporting lower resilience. Evidence from the included literature suggests that lower resilience in BD was associated with, but not limited to, the following areas: increased rumination [43], lower social functioning [47], greater impulsivity [52], fearful attachment style [51], worse family functioning [57], and childhood trauma [49, 50] suggesting that resilience is affected in various domains. To further inspect these results, we analyzed resilience subdomains, finding significant differences, with patients with BD reporting lower scores in all subdomains except for spiritual influences. Specifically, two items (3 and 9 respectively) of the CD‐RISC measure spiritual influences. We propose some limitations to these items to interpret why this is the only subdomain that did not show significant differences. If we ask a person with BD to rate Item 3 “Sometimes fate or God can help me” they may respond lower, as the consequence of fate and/or God has been to diagnose them with a chronic condition. Conversely, this item could also be problematic in that patients with BD may believe that fate/God can help them find a cure, which to date does not exist; thus, they may overestimate their response. Similarly, item 9 “Things happen for a reason” could pose problems for patients with BD, as it may be difficult to attribute BD happening for a reason, as well as the fact that it would be dependent on what “thing” happens for a reason. For example, missing the bus, experiencing a break‐up, not receiving a job offer, and experiencing a manic episode are all “things” that may happen for a “reason” but the impact upon a person's life of each event differs greatly. Further, spiritual influences may be a source of resilience independent of mental status, which could also be linked to cultural/spiritual background. Finally, perhaps the items are not sensitive enough to the context of SMI, as they do not take into account the spiritual needs of people with BD, which may differ from the general population. As such, in people with BD, items referring to spirituality, such as fate or God, may be especially sensitive, given the chronicity of BD, as well as the heterogeneity of each individual experience, which could be influenced by illness experience, differing from the way spirituality is captured in the general population. This underscores the need to account for cultural and spiritual frameworks when interpreting responses to ensure measurement tools reflect the varied ways people perceive support. Nevertheless, only two of the included studies [50, 51] analyzed resilience at the subdomain level; thus, we urge caution against drawing definitive conclusions in BD.

Subsequent meta‐regression analyses revealed that euthymia was associated with fewer differences in resilience between BD and HCs, albeit BD still reported lower resilience scores. This result could be of clinical importance as it highlights the potential need to consider resilience as part of the long‐term treatment plan given its potential fluctuations. If resilience does indeed fluctuate, the timing of specific interventions could also be an important factor to take into account. For example, clinicians might prioritize resilience‐focused therapies during the euthymic phase to consolidate coping skills and improve long‐term prognosis. While this indicates that periods of euthymia are associated with feelings of greater resilience, small effect sizes were found and only four studies were included in this analysis, potentially limiting statistical power and generalizability of results. Nevertheless, the lower resilience scores between BD and HCs may be best interpreted in the broader context of illness‐related burden. Two interpretations are plausible. First, persistent cognitive and functional impairments, even during euthymia [8, 9], may undermine patients' sense of resilience. Second, unlike individuals with BD, HCs do not contend with ongoing affective symptoms, which may inflate their perceived resilience. This highlights a key limitation in using HCs as controls in resilience studies. For instance, depression is linked to reduced resilience [76], suggesting that the absence of such episodes in HCs may partly explain group differences. Future research should address these methodological issues and incorporate mood state as a critical variable when studying resilience across illness phases in BD.

Importantly, high heterogeneity was observed in the BD and HC comparison. This could be explained by the various assessment tools used to examine resilience, as well as the differences in operationalizing resilience across included studies. Consequently, our findings echo the questions raised in the ongoing debate regarding the definition of resilience, specifically in BD. If, by definition, greater resilience refers to the absence of psychopathology or maintenance of mental health, then greater/lower resilience will be defined as mental illness/no mental illness, and, in turn, people with SMI will report feeling less resilient than their HC counterparts [77]. A further, and notably more negative consequence, is the risk of labelling people with mental illness as “not resilient” or “less resilient” due to the presence of the disorder itself. This could increase stigma, which has been associated with greater functional impairment, symptoms of depression and anxiety, and barriers to treatment seeking in BD [78]. As such, could it be that patients with BD are not *less* resilient than HCs per se, but they *feel* less resilient given illness impact in their daily lives within the specific context of SMI, which HCs do not experience? Accordingly, the dichotomic approach to resilience in BD may be replaced by considering resilience as a multidimensional characteristic to provide greater insight into the intricacies of this complex construct [18, 79, 80]. Future research should adopt a more consistent, consensus‐driven approach to defining and measuring resilience in BD. This would allow for a clearer, more inclusive understanding of resilience in this population and help identify strategies to enhance resilience within psychological interventions.

### 4.2 Resilience in BD and Clinical Populations

The absence of significant differences in reported resilience between BD and MDD and high heterogeneity could be related to the symptom overlap between these mood disorders. Depressive symptoms represent the most frequent polarity in BD [81] with long‐term subsyndromal symptoms often present [82]. In addition, depression has also been associated with reduced resilience [76]. In the context of BD, bipolar depression represents an unresolved clinical challenge and is strongly associated with disability and higher patient mortality rates [83]. Moreover, misdiagnosis of BD as MDD is common in approximately 40% of patients [84] and an average delay of 6–8 years for receiving a correct diagnosis and treatment has also been reported [85]. As such, challenges regarding diagnosis, mood state, and associated clinical symptomatology may have limited the possibility of finding differences in resilience between BD and MDD. Future research is thus required to provide greater insight into the relationship between resilience and depressive symptoms in BD and MDD.

Conversely, significant differences were observed for BD and SCZ, with BD reporting greater resilience. All five studies included in this analysis used cross‐sectional data, limiting interpretations of associations between variables of interest, underlining a need for longitudinal research to gain further insight into the dynamic process of resilience. Nonetheless, one possible factor explaining the observed differences could be cognitive impairment. Cognitive impairment in BD and SCZ predicts poor functional outcomes [86, 87, 88] thus potentially impacting patients' ability when self‐reporting subjective resilience. Moreover, comparing cognitive impairment can be limited by methodological difficulties in sample characteristics. Only one study [55] investigated cognitive impairment and resilience in BD, SCZ, and HCs and found that SCZ had greater cognitive impairment and lower resilience than BD. These findings could be influenced by the DOI, linked to increased cognitive impairment in BD and SCZ. Specifically, two studies [45, 62] reported longer DOI in SCZ patients which, in turn, we suggest could impact resilience as the illness progresses. This is supported by a recent meta‐analysis from Bortolato et al. [83] eluding that cognitive impairment is generally more severe in SCZ than BD, while also suggesting caution as results appear to be non‐conclusive, highlighting a potential need to explore the severity of cognitive impairment in early illness onset of both disorders [89]. Accordingly, people with greater DOI and cognitive impairment may benefit from specific interventions including a combination of cognitive and resilience training. Similarly, resilience interventions at early illness onset or for people identified as high risk could also be used to improve long‐term prognosis, increasing resilience before reaching chronic stages. Improved methodological approaches using longitudinal designs are required to explore the potential relationship between DOI, cognitive impairment, and resilience in BD and SCZ.

### 4.3 Assessments of Resilience

In terms of assessing resilience, all articles in the present study used self‐report scales to measure resilience in SMI. Across studies, eight different resilience measurement scales, representing different theoretical standpoints on the construct, were used. Self‐report tools are vulnerable to bias such as social desirability, acquiescence, affective bias, and reduced insight [5, 90]. Although these scales are the most widely used in resilience research, they depend on a person's ability to self‐assess their resilience in an adverse situation and could represent important limitations in a clinical setting. This is particularly evident in BD as patients are likely to recognise symptoms of depression while minimising and/or ignoring hypo(manic) features of the disorder [1]. It is plausible, therefore, that mood state affects insight regarding both illness features and resilience. Despite its relevance, only 11 of the 21 identified studies in this review provided information on BD mood state. To counteract these challenges, we suggest a need for clinician‐rated assessments of resilience to offer more objective measures of resilience, vital when changing mood states are likely to affect subjective self‐interpretation and insight. Such an instrument could be used at different clinical stages in BD (hypo)mania, depressed, and euthymic to validate its efficacy in this population. At the same time, this reiterates that more strict reporting criteria on mood state are required to adopt a universal approach to understanding the effects of different stages of BD, a disorder characterized by mood disturbances.

Furthermore, concerns regarding the validation of the identified scales in people with a mental health diagnosis were observed. For example, in their original study, the RSA [74] included a clinical population with mental health diagnoses, which included BD (*n* = 2), SCZ (*n* = 1), and MDD (*n* = 24); however, the factor analysis did not include the psychiatric sample. The RBD [60] was developed for the measurement of resilience in BD only, restricting its usage in the broader context of SMI. Also, the CD‐RISC [71] did not include the psychopathology groups with modest sample sizes in the main internal consistency validity analysis, opting to include them in the test–retest reliability analysis and some other subgroup analyses. No other scale was specifically designed for use in people with clinically diagnosed mental health disorders to the best of our knowledge. This highlights a core clinical challenge in ensuring that the recommended and widely used measurements are representative of resilience and offer enough sensitivity to capture changes within specific target populations such as BD and SMI [91]. Moreover, differences within samples must also be considered as demonstrated by Montejo et al. [69], who used an adapted version of the CD‐RISC for older age bipolar disorder (OABD) given their unique characteristics as a potential “survivor cohort” [92]. In addition, self‐report scales may contribute to the challenges of dichotomy in the definition of resilience by establishing cut‐off points, established via heterogeneous non‐SMI populations. Given that there is an ongoing debate regarding the definition and measurement of resilience, we suggest the need to develop a specific assessment tool for resilience in the SMI population in order to avoid the potential confounding effects associated with using an array of different measurements. This could drive research towards finding a *gold standard* assessment tool, with higher transferability for the effective measurement of resilience in SMI. If this issue is not addressed, the potential to compromise content validity in the measurement of resilience remains high.

### 4.4 Limitations

Several limitations of the present meta‐analysis must be acknowledged. Significant heterogeneity limited the generalizability of findings, though sensitivity analyses were conducted. A standardized definition and assessment of resilience is needed to improve consistency across studies. Most included studies were cross‐sectional, limiting causal inferences [90] and the small sample size [93] may affect robustness. Furthermore, the role of sex differences and onset age remain unclear. This is of importance given that females represented a larger proportion of the BD (60.9%) and MDD (72.9%) samples, but less of the SCZ (40.7%) sample. Similarly, differences in onset age between sexes could also be considered as MDD had a later onset age (*M* = 39.14) than both BD (*M* = 25.68) and SCZ (*M* = 24.3). Moreover, in our meta‐regression analyses (Table S4), neither sex distribution nor age at onset emerged as significant moderators of the results. Thus, while these factors did not explain the heterogeneity in the present study, they could still influence resilience trajectories and should be carefully examined in future research. As results on both aspects remain inconclusive [94, 95], and could be influencing the differences in results, we suggest that future research aims to recruit more inclusive, balanced samples taking these potential differences into account. The exclusive use of quantitative data may overlook important lived experiences of resilience in SMI, which qualitative research could help capture. A person‐centred approach may also enhance resilience by involving patients in shaping recovery goals [96]. Data on mood state, medication use, and psychotherapy were limited, despite their potential influence on resilience [97]. Finally, reliance on self‐report underscores the need for more objective, ecologically valid measures. Despite these limitations, this study advances the field by including a meta‐analysis, specifying diagnostic criteria, and broadening inclusion beyond English‐language studies, addressing gaps in prior reviews Chan et al. [31].

## 5 Conclusions

The current systematic review and meta‐analysis sought to review the available evidence on resilience in BD compared to clinical and non‐clinical populations. Overall, the findings showed that BD reports less resilience than HCs, similar resilience to MDD, and greater resilience than SCZ. Moreover, no differences were found in the comparisons between BD and MDD. A key limitation across all studies was the exclusive reliance on self‐report measures, underscoring the need for more robust assessment methods, such as clinician‐administered interviews tailored to BD and SMI populations.

These findings must be interpreted within the context of ongoing methodological challenges in resilience research. Inconsistencies in the definition, operationalization, and measurement of resilience continue to hinder clarity and generalizability. Our results align with previous calls for improved conceptual and methodological standards in resilience research, particularly within psychiatric populations. Differentiating resilience profiles between clinical and non‐clinical groups holds important implications for both research and clinical practice. Resilience‐enhancing strategies could inform prevention efforts and support recovery in those with lived experience of mental illness. Future studies should prioritize longitudinal designs to track resilience across the illness course [98], explore key clinical variables (e.g., mood state, depressive symptoms, duration of illness), and examine links between resilience, cognitive impairment, and psychosocial functioning in BD. A multidisciplinary approach is essential to advance the field and inform interventions aimed at promoting resilience in BD and other SMI populations—ultimately helping to reduce the long‐term burden of these chronic conditions, allowing people to continue to live well within the context of their mental health diagnosis.

## Author Contributions

D.C., S.A., C.T., designed the study, wrote the protocol, conducted article screening, and data extraction. M.D.P. conducted the literature searches and the statistical analysis. B.S. and L.M. contributed to the article screening and preparation of the Supporting Information. C.T. and S.A. contributed to article screening. D.C. wrote the first draft of the manuscript. M.S.‐N., M.F.F., P.C.‐B., E.d.l.S., V.O., S.M.‐P., J.S.‐M., B.L.A., M.G., N.V., M.R., A.M.‐A., E.V. revised the manuscript at various stages. D.C., S.A., C.T., M.D.P., B.S., L.M., M.S.‐N., M.F.F., P.C.‐B., E.d.l.S., V.O., S.M.‐P., J.S.‐M., B.L.A., M.G., N.V., M.R., A.M.‐A., E.V. contributed to and approved the final manuscript.

## Conflicts of Interest

The authors declare no conflicts of interest.

## Supporting information


**Appendix S1:** Supporting Information.

## Data Availability

All data generated or analyzed during this study are included in this article. Further enquiries can be directed to the corresponding author. Links to specific data are also available in the Supporting Information as indicated accordingly.
