# Supplementary material for: Resilience in Bipolar Disorder Compared to Clinical and Non‐Clinical Populations: A Systematic Review and Meta‐Analysis
Source: Acta Psychiatr Scand. 2025 Nov 12;153(1):6–23. doi: 10.1111/acps.70042 (PMC12668893; doi:10.1111/acps.70042)
Supplement: Supplementary file 1 — Appendix S1: Supporting Information. [file ACPS-153-6-s001.docx]

Table of Contents

[APPENDIX I 3](#_Toc196404435)

[PRISMA Checklist 3](#_Toc196404436)

[APPENDIX II 6](#_Toc196404437)

[Deviations from the original protocol 6](#_Toc196404438)

[APPENDIX III 7](#_Toc196404439)

[Search strategy 7](#_Toc196404440)

[APPENDIX IV 8](#_Toc196404441)

[Excluded studies, with reasons 8](#_Toc196404442)

[APPENDIX V 12](#_Toc196404443)

[Quality according to the Newcastle-Ottawa Scale of included cross-sectional studies 12](#_Toc196404444)

[APPENDIX VI 14](#_Toc196404445)

[Main analysis, two-groups meta-analysis 14](#_Toc196404446)

[Meta-regression analyses, two-groups meta-analysis 15](#_Toc196404447)

[Sensitivity analyses, leave-one-out sensitivity analysis, two-groups meta-analysis 17](#_Toc196404448)

[Main analysis, single-group meta-analysis 19](#_Toc196404449)

[Meta-regression analyses, single-group meta-analysis 20](#_Toc196404450)

[Sensitivity analyses, leave-one-out sensitivity analysis, single-group meta-analysis 22](#_Toc196404451)

[APPENDIX VII 24](#_Toc196404452)

[Publication bias, two-groups meta-analysis 24](#_Toc196404453)

[Publication bias, single-group meta-analysis 25](#_Toc196404454)

# APPENDIX I

## PRISMA Checklist

**Table 1 - PRISMA Checklist**

| **1Section and Topic** | **Item #** | **Checklist item** | **Location where item is reported** |
| --- | --- | --- | --- |
| **TITLE** | | |  |
| Title | 1 | Identify the report as a systematic review. | Page 1 |
| **ABSTRACT** | | |  |
| Abstract | 2 | See the PRISMA 2020 for Abstracts checklist. | Page 2 |
| **INTRODUCTION** | | |  |
| Rationale | 3 | Describe the rationale for the review in the context of existing knowledge. | Page 5 |
| Objectives | 4 | Provide an explicit statement of the objective(s) or question(s) the review addresses. | Page 5 |
| **METHODS** | | |  |
| Eligibility criteria | 5 | Specify the inclusion and exclusion criteria for the review and how studies were grouped for the syntheses. | Page 5 |
| Information sources | 6 | Specify all databases, registers, websites, organisations, reference lists and other sources searched or consulted to identify studies. Specify the date when each source was last searched or consulted. | Page 6 |
| Search strategy | 7 | Present the full search strategies for all databases, registers and websites, including any filters and limits used. | Supp Matt |
| Selection process | 8 | Specify the methods used to decide whether a study met the inclusion criteria of the review, including how many reviewers screened each record and each report retrieved, whether they worked independently, and if applicable, details of automation tools used in the process. | Page 6 |
| Data collection process | 9 | Specify the methods used to collect data from reports, including how many reviewers collected data from each report, whether they worked independently, any processes for obtaining or confirming data from study investigators, and if applicable, details of automation tools used in the process. | Page 6 |
| Data items | 10a | List and define all outcomes for which data were sought. Specify whether all results that were compatible with each outcome domain in each study were sought (e.g. for all measures, time points, analyses), and if not, the methods used to decide which results to collect. | Page 7 |
|  | 10b | List and define all other variables for which data were sought (e.g. participant and intervention characteristics, funding sources). Describe any assumptions made about any missing or unclear information. | Page 7 |
| Study risk of bias assessment | 11 | Specify the methods used to assess risk of bias in the included studies, including details of the tool(s) used, how many reviewers assessed each study and whether they worked independently, and if applicable, details of automation tools used in the process. | Page 10 |
| Effect measures | 12 | Specify for each outcome the effect measure(s) (e.g. risk ratio, mean difference) used in the synthesis or presentation of results. | Page 9 |
| Synthesis methods | 13a | Describe the processes used to decide which studies were eligible for each synthesis (e.g. tabulating the study intervention characteristics and comparing against the planned groups for each synthesis (item #5)). | Page 7 |
|  | 13b | Describe any methods required to prepare the data for presentation or synthesis, such as handling of missing summary statistics, or data conversions. | Page 7 |
|  | 13c | Describe any methods used to tabulate or visually display results of individual studies and syntheses. | Page 9 |
|  | 13d | Describe any methods used to synthesize results and provide a rationale for the choice(s). If meta-analysis was performed, describe the model(s), method(s) to identify the presence and extent of statistical heterogeneity, and software package(s) used. | Page 9 |
|  | 13e | Describe any methods used to explore possible causes of heterogeneity among study results (e.g. subgroup analysis, meta-regression). | Page 7 |
|  | 13f | Describe any sensitivity analyses conducted to assess robustness of the synthesized results. | Page 10 |
| Reporting bias assessment | 14 | Describe any methods used to assess risk of bias due to missing results in a synthesis (arising from reporting biases). | Page 10 |
| Certainty assessment | 15 | Describe any methods used to assess certainty (or confidence) in the body of evidence for an outcome. | Page 6 |
| **RESULTS** | | |  |
| Study selection | 16a | Describe the results of the search and selection process, from the number of records identified in the search to the number of studies included in the review, ideally using a flow diagram. | Page 7-9 |
|  | 16b | Cite studies that might appear to meet the inclusion criteria, but which were excluded, and explain why they were excluded. | N/A |
| Study characteristics | 17 | Cite each included study and present its characteristics. | Table 1  Page 7-8 |
| Risk of bias in studies | 18 | Present assessments of risk of bias for each included study. | Supp Matt |
| Results of individual studies | 19 | For all outcomes, present, for each study: (a) summary statistics for each group (where appropriate) and (b) an effect estimate and its precision (e.g. confidence/credible interval), ideally using structured tables or plots. | Page 7-8  Fig. 2 |
| Results of syntheses | 20a | For each synthesis, briefly summarise the characteristics and risk of bias among contributing studies. | Table 1 |
|  | 20b | Present results of all statistical syntheses conducted. If meta-analysis was done, present for each the summary estimate and its precision (e.g. confidence/credible interval) and measures of statistical heterogeneity. If comparing groups, describe the direction of the effect. | Page 7-9 Supp Mat |
|  | 20c | Present results of all investigations of possible causes of heterogeneity among study results. | Page 9 |
|  | 20d | Present results of all sensitivity analyses conducted to assess the robustness of the synthesized results. | Page 8-9, Supp Mat |
| Reporting biases | 21 | Present assessments of risk of bias due to missing results (arising from reporting biases) for each synthesis assessed. | Page10 |
| Certainty of evidence | 22 | Present assessments of certainty (or confidence) in the body of evidence for each outcome assessed. | Table 1 |
| **DISCUSSION** | | |  |
| Discussion | 23a | Provide a general interpretation of the results in the context of other evidence. | Page 10-16 |
|  | 23b | Discuss any limitations of the evidence included in the review. | Page 14-15 |
|  | 23c | Discuss any limitations of the review processes used. | Page 14-15 |
|  | 23d | Discuss implications of the results for practice, policy, and future research. | Page 16 |
| **OTHER INFORMATION** | | |  |
| Registration and protocol | 24a | Provide registration information for the review, including register name and registration number, or state that the review was not registered. | Page 5 |
|  | 24b | Indicate where the review protocol can be accessed, or state that a protocol was not prepared. | Page 5 |
|  | 24c | Describe and explain any amendments to information provided at registration or in the protocol. | Page 5 |
| Support | 25 | Describe sources of financial or non-financial support for the review, and the role of the funders or sponsors in the review. | Page 17 |
| Competing interests | 26 | Declare any competing interests of review authors. | Page 17 |
| Availability of data, code and other materials | 27 | Report which of the following are publicly available and where they can be found: template data collection forms; data extracted from included studies; data used for all analyses; analytic code; any other materials used in the review. | Supp Matt |

# APPENDIX II

## Deviations from the original protocol

In the original protocol, we planned to perform meta-regressions only for meta-analyses including at least ten studies. However, to better understand the role of different predictors in our results, we decided to conduct exploratory meta-regressions whenever high heterogeneity was detected.

# APPENDIX III

## Search strategy

**PubMed/MEDLINE (up to 08.08.24: 373 results)**

("bipolar disorder"[Title/Abstract] OR "mania"[Title/Abstract] OR "manic"[Title/Abstract] OR "hypomania"[Title/Abstract] OR "hypomanic"[Title/Abstract] OR "bipolar depression"[Title/Abstract] OR "bipolar disorder"[MeSH Terms] OR "affective disorders, psychotic"[MeSH Terms]) AND ("resilien*"[Title/Abstract] OR "psychological resilien*"[Title/Abstract] OR "resilience, psychological"[MeSH Terms])

**Scopus (up to 08.08.24: 499 results)**

(TITLE-ABS("bipolar disorder") OR TITLE-ABS(mania) OR TITLE-ABS(manic) OR TITLE-ABS(hypomania) OR TITLE-ABS(hypomanic) OR TITLE-ABS("bipolar depression") OR INDEXTERMS("bipolar disorder") OR INDEXTERMS("affective disorders, psychotic")) AND (TITLE-ABS(resilien*) OR TITLE-ABS("psychological resilien*") OR INDEXTERMS("resilience, psychological"))

**PsycINFO (up to 08.08.24: 297 results)**

((TI "bipolar disorder" OR AB "bipolar disorder") OR (TI mania OR AB mania) OR (TI manic OR AB manic) OR (TI hypomania OR AB hypomania) OR (TI hypomanic OR AB hypomanic) OR (TI "bipolar depression" OR AB "bipolar depression") OR (SU "bipolar disorder") OR (SU "affective psychotic")) AND ((TI resilien* OR AB resilien*) OR (TI "psychological resilien*" OR AB "psychological resilien*") OR (SU "Resilience (Psychological)"))

# APPENDIX IV

## Excluded studies, with reasons

**Table 2 - Excluded studies with reasons**

| **Author, year** | **Title** | **doi** | **Exclusion reason** |
| --- | --- | --- | --- |
| Acar Sivri et al. 2019 | Resilience and personality in pschiatric patients | <https://doi.org/10.1080/24750573.2018.1540199> | Wrong population |
| Adachi et al. 2020 | Characteristics of university students supported by counselling services: Analysis of psychological tests and pulse rate variability | [10.1371/journal.pone.0218357](https://doi.org/10.1371/journal.pone.0218357) | Wrong population |
| Aguilar et al. 2020 | Positive sports psychiatry | Unavailable | Wrong publication |
| Aksoy et al. 2022 | Clinical phenomenology in children and adolescents with bipolar disorder | Unavailable |  |
| Belz et al. 2022 | Evolution of psychosocial burden and psychiatric symptoms in patients with psychiatric disorders during the Covid-19 pandemic | https://doi.org/10.1007/s00406-021-01268-6 | Wrong outcome |
| Camardese et al. 2018 | A mixed program of psychoeducational and psychological rehabilitation for patients with bipolar disorder in a day hospital setting | 10.1097/NMD.0000000000000795 | Wrong outcome |
| Chandra et al. 2022 | Basic functioning and resilience in families of adolescents with intellectual disability and psychosis: A comparative study | 10.4103/ijsp.ijsp_209_20 | Wrong outcome |
| Chen et al. 2018 | Resilience of functional networks: A potential indicator for classifying bipolar disorder and schizophrenia | [10.1109/CACS.2017.8284247](https://doi.org/10.1109/CACS.2017.8284247) | Wrong outcome |
| Chin Fatt et al. 2021 | Dysfunction of default mode network is associated with active suicidal ideation in youths and young adults with depression: Findings from the T-RAD study | <https://doi.org/10.1016/j.jpsychires.2021.07.047> | Wrong outcome |
| Chung et al. 2017 | An examination of resilience in the Longitudinal Assessment of Manic Symptoms study | Unavailable | Wrong publication type |
| Citak & Erten. 2021 | Impact of childhood trauma and attachment on resilience in remitted patients with bipolar disorder | <https://doi.org/10.1016/j.jad.2020.11.025> | Wrong outcome |
| Colpe et al. 2015 | Mental health treatment among soldiers with current mental disorders in the Army Study to Assess Risk and Resilience in Service members (Army STARRS) | <https://doi.org/10.7205/MILMED-D-14-00686> | Wrong outcome |
| Dima et al. 2016 | Connectomic markers of disease expression, genetic risk and resilience in bipolar disorder | https://doi.org/10.1038/tp.2015.193 | Wrong outcome |
| Echezarraga et al. 2018 | Resilience dimensions and mental health outcomes in bipolar disorder in a follow-up study | <https://doi.org/10.1002/smi.2767> | Wrong outcome |
| Echezarraga et al. 2022 | Resilience moderates the associations between bipolar disorder mood episodes and mental health | <https://doi.org/10.5093/clysa2022a8> | Wrong outcome |
| Echezarraga et al. 2019 | Resilience process in bipolar disorder from the views of patients and health professionals | 10.24205/03276716.2019.1125 | Wrong outcome |
| Fernández-Rocha et al. 2021 | Psychological Resilience and Suicide Attempt in Patients With Bipolar Disorder: An Exploratory Study | https://doi.org/10.1177/10783903211050682 | Wrong outcome |
| Gutierrez et al. 2023 | Evaluating the Efficacy of Web-Based Cognitive Behavioral Therapy for the Treatment of Patients With Bipolar II Disorder and Residual Depressive Symptoms: Protocol for a Randomized Controlled Trial | [10.2196/46157](https://doi.org/10.2196/46157) | Wrong publication type |
| Hiyoshi et al. 2017 | Precursors in adolescence of adult-onset bipolar disorder | <https://doi.org/10.1016/j.jad.2017.04.071> | Wrong outcome |
| Hofer et al. 2022 | Mental health in individuals with severe mental disorders during the covid-19 pandemic: a longitudinal investigation | https://doi.org/10.1038/s41537-022-00225-z | Wrong outcome |
| Karimirad et al. 2018 | The relationship between resilience and quality of life in family caregivers of patients with mental disorders | <https://doi.org/10.7860/jcdr/2018/29369.12239> | Wrong population |
| Kempton et al. 2009 | Dissociable brain structural changes associated with predisposition, resilience, and disease expression in bipolar disorder | https://doi.org/10.1523/JNEUROSCI.2204-09.2009 | Wrong outcome |
| Kessler et al. 2009 | Thirty-day prevalence of dsm-iv mental disorders among nondeployed soldiers in the us army results from the army study to assess risk and resilience in servicemembers (army starrs) | 10.1001/jamapsychiatry.2014.28 | Wrong outcome |
| Liu et al. 2022 | The experiences of family resilience from the view of the adult children of parents with bipolar disorder in Chinese society | <https://doi.org/10.1111/jan.15008> | Wrong outcome |
| Luther et al. 2020 | The multidimensional construct of resilience across the psychosis spectrum: Evidence of alterations in people with early and prolonged psychosis | [https://doi.org/10.1037/prj0000393](https://psycnet.apa.org/doi/10.1037/prj0000393) | Wrong population |
| Lytaev et al. 2023 | Psychological and Neurophysiological Screening Investigation of the Collective and Personal Stress Resilience | <https://doi.org/10.3390/bs13030258> | Wrong outcome |
| Macoveanu et al. 2021 | Hippocampal subfield morphology in monozygotic twins discordant for affective disorders | https://doi.org/10.1038/s41386-020-0756-2 | Wrong outcome |
| Mizuno et al. 2018 | Religiosity and psychological resilience in patients with schizophrenia and bipolar disorder: an international cross-sectional study | <https://doi.org/10.1111/acps.12838> | Wrong outcome |
| Montejo et al. 2021 | Self-reported neurocognitive symptoms during COVID-19 lockdown and its associated factors in a sample of psychiatric patients Results from the BRIS-MHC study | <https://doi.org/10.1016/j.euroneuro.2021.07.006> | Wrong outcome |
| Nishi et al. 2013 | Culturally sensitive and universal measure of resilience for Japanese populations: Tachikawa Resilience Scale in comparison with Resilience Scale 14-item version | <https://doi.org/10.1111/pcn.12028> | Wrong outcome |
| Orhan et al. 2022 | The course of psychiatric symptoms in older age bipolar disorder during the COVID-19 pandemic | https://doi.org/10.1186/s40345-022-00274-4 | Wrong outcome |

# APPENDIX V

## Quality according to the Newcastle-Ottawa Scale of included cross-sectional studies

**Table 3 - Detailed description of the quality of the included cross-sectional studies according to the Newcastle-Ottawa Scale**

| **Author, year** | **Representativeness of the sample (SELECTION)** | **Sample Size (SELECTION)** | **Non-respondents (SELECTION)** | **Ascerteinment of the exposure (SELECTION)** | **Comparability (COMPARABILITY)** | **Assessment of the outcome (OUTCOME)** | **Statistical Test (OUTCOME)** | **TOTAL** | **AHRQ Standards** |
| --- | --- | --- | --- | --- | --- | --- | --- | --- | --- |
| Aslan et al. 2005 | 1 | 0 | 1 | 2 | 2 | 2 | 1 | 9 | Very good |
| Bozikas et al. 2018 | 1 | 0 | 0 | 2 | 2 | 2 | 1 | 8 | Good |
| Chiang et al. 2024 | 1 | 0 | 0 | 2 | 0 | 1 | 1 | 5 | Satisfactory |
| Choi et al. 2015 | 1 | 0 | 0 | 2 | 2 | 2 | 1 | 8 | Good |
| Chung et al. 2018 | 0 | 0 | 1 | 2 | 2 | 2 | 1 | 8 | Good |
| Craba et al. 2023 | 1 | 0 | 0 | 2 | 2 | 2 | 1 | 8 | Good |
| Datta & Chetia. 2023 | 1 | 1 | 0 | 2 | 1 | 2 | 1 | 8 | Good |
| Deng et al. 2018 | 1 | 1 | 0 | 2 | 0 | 2 | 1 | 7 | Good |
| Dou et al. 2021 | 0 | 0 | 0 | 2 | 2 | 2 | 1 | 7 | Good |
| Echezarraga et al. 2017 | 1 | 0 | 1 | 2 | 2 | 2 | 1 | 9 | Very good |
| Favale et al. 2023 | 1 | 0 | 0 | 2 | 2 | 2 | 1 | 8 | Good |
| Hofer et al. 2017 | 1 | 0 | 0 | 2 | 2 | 1 | 1 | 7 | Good |
| Just et al. 2022 | 1 | 0 | 0 | 2 | 1 | 1 | 1 | 6 | Good |
| Kang et al. 2024 | 1 | 0 | 0 | 2 | 0 | 1 | 1 | 5 | Satisfactory |
| Kesebir et al. 2015 | 1 | 0 | 0 | 2 | 0 | 1 | 1 | 5 | Satisfactory |
| Lee et al. 2017 | 1 | 0 | 0 | 2 | 2 | 1 | 1 | 7 | Good |
| Mackali et al. 2023 | 1 | 1 | 1 | 2 | 0 | 2 | 1 | 8 | Very good |
| Mizuno et al. 2016 | 1 | 1 | 1 | 2 | 1 | 2 | 1 | 9 | Very good |
| Montejo et al. 2024 | 1 | 0 | 0 | 2 | 0 | 2 | 1 | 6 | Satisfactory |
| Nunes et al. 2022 | 1 | 0 | 0 | 2 | 1 | 1 | 1 | 6 | Satisfactory |
| Palagini et al. 2022 | 1 | 0 | 0 | 1 | 0 | 2 | 1 | 5 | Satisfactory |
| Park et al. 2023 | 1 | 0 | 0 | 2 | 2 | 1 | 1 | 7 | Good |
| Post et al. 2018 | 1 | 1 | 0 | 2 | 0 | 2 | 1 | 7 | Very good |
| Sato et al. 2023 | 1 | 0 | 0 | 2 | 2 | 2 | 1 | 8 | Very good |
| Şenormanci et al. 2020 | 1 | 0 | 0 | 2 | 1 | 1 | 1 | 6 | Satisfactory |
| Tsigkaropoulou et al. 2023 | 1 | 0 | 0 | 2 | 2 | 2 | 1 | 8 | Good |
| Uygun et al. 2020 | 1 | 0 | 0 | 2 | 2 | 2 | 1 | 8 | Good |
| Vieira et al. 2020 | 1 | 0 | 1 | 2 | 2 | 2 | 1 | 9 | Very good |

#

# APPENDIX VI

## Main analysis, two-groups meta-analysis

The forest plots for each meta-analysis are available at https://osf.io/qxh3y/?view_only=d0f7414073fe41c08b5f595a81ed33b4.

##

## Meta-regression analyses, two-groups meta-analysis

**Table 4 - Meta-regression analyses**

The results are highlighted in green when a higher value of that specific predictor is significantly associated with a larger magnitude difference between the two groups.

The results are highlighted in blue when a higher value of that specific predictor is significantly associated with a smaller magnitude difference between the two groups.

| **Control group** | **Outcome type** | **Predictor** | **Studies, n** | **Beta** | **95% CIs** | **p-value** | **SMD at predictor, lower value** | **SMD at predictor, higher value** |
| --- | --- | --- | --- | --- | --- | --- | --- | --- |
| HCs | Total | Mean age | 16 | -0.002 | -0.024, 0.02 | 0.86 | -0.781 at 22.71 | -0.84 at 51.3 |
| HCs | Total | Percentage of females | 16 | -0.012 | -0.029, 0.005 | 0.17 | -0.623 at 44.1 | -0.994 at 75 |
| HCs | Total | Percentage of people with BD1 | 11 | -0.004 | -0.01, 0.002 | 0.17 | -0.529 at 0 | -0.942 at 100 |
| HCs | Total | Duration of illness | 10 | 0.007 | -0.016, 0.031 | 0.53 | -0.799 at 7.56 | -0.572 at 38.13 |
| HCs | Total | Age at onset | 8 | 0.005 | -0.081, 0.091 | 0.91 | -0.755 at 21.22 | -0.689 at 34.1 |
| HCs | Total | MADRS symptoms severity | 6 | -0.006 | -0.062, 0.05 | 0.83 | -0.799 at 3.8 | -0.848 at 12 |
| HCs | Total | YMRS symptoms severity | 6 | 0.031 | -0.053, 0.115 | 0.47 | -0.876 at 1.04 | -0.628 at 9.06 |
| HCs | Total | Percentage of people in euthymia | 4 | 0.007 | 0.001, 0.014 | 0.022 | -1.682 at 0 | -0.946 at 100 |
| MDD | Total | Percentage of females | 9 | 0.021 | -0.012, 0.053 | 0.21 | -0.291 at 47.3 | 0.244 at 73.3 |
| MDD | Total | Mean age | 8 | 0.006 | -0.026, 0.038 | 0.72 | -0.02 at 25.78 | 0.248 at 71 |
| MDD | Total | Duration of illness | 4 | -0.179 | -0.363, 0.005 | 0.06 | 0.586 at 8.91 | -0.557 at 15.3 |
| MDD | Total | Percentage of people with BD1 | 4 | 0.009 | 0.003, 0.015 | 0.002 | 0.005 at 22.1 | 0.429 at 67.5 |
| MDD | Total | Age at onset | 3 | 0.008 | -0.144, 0.16 | 0.92 | 0.353 at 26.2 | 0.386 at 30.2 |
| MDD | Total | MADRS symptoms severity | 3 | -0.065 | -0.154, 0.024 | 0.15 | -0.084 at 12 | -0.802 at 23 |
| SCZ | Total | Mean age | 5 | -0.013 | -0.041, 0.014 | 0.35 | 0.553 at 22.71 | 0.188 at 50.2 |
| SCZ | Total | Percentage of females | 5 | -0.007 | -0.037, 0.023 | 0.65 | 0.46 at 36.7 | 0.245 at 67.6 |
| SCZ | Total | Duration of illness | 4 | 0.021 | -0.003, 0.045 | 0.09 | 0.19 at 11 | 0.755 at 38.13 |
| SCZ | Total | MADRS symptoms severity | 3 | 0.048 | -0.154, 0.25 | 0.64 | 0.2 at 4.6 | 0.556 at 12 |
| SCZ | Total | YMRS symptoms severity | 3 | 0.068 | -0.062, 0.198 | 0.3 | 0.182 at 1.2 | 0.718 at 9.06 |

## Sensitivity analyses, leave-one-out sensitivity analysis, two-groups meta-analysis

**Table 5 - Leave-one-out sensitivity analysis**

The results are highlighted in red when the removal of that particular study changes a previously significant association to not significant.

| **Control group** | **Outcome type** | **Author, year of the study removed** | **SMD** | **95% CIs** | **p-value** | **I2** | **tau2** | **Q test p-value** |
| --- | --- | --- | --- | --- | --- | --- | --- | --- |
| HCs | Total | Aslan et al. 2005 | -0.739 | -0.877, -0.601 | <0.001 | 76.73 | 0.06 | <0.1 |
| HCs | Total | Bozikas et al. 2018 | -0.777 | -0.939, -0.615 | <0.001 | 83.72 | 0.09 | <0.1 |
| HCs | Total | Choi et al. 2015 | -0.788 | -0.953, -0.622 | <0.001 | 84.08 | 0.09 | <0.1 |
| HCs | Total | Chung et al. 2018 | -0.816 | -0.971, -0.66 | <0.001 | 80.83 | 0.08 | <0.1 |
| HCs | Total | Craba et al. 2023 | -0.786 | -0.951, -0.621 | <0.001 | 84.08 | 0.09 | <0.1 |
| HCs | Total | Deng et al. 2018 | -0.799 | -0.962, -0.635 | <0.001 | 83.86 | 0.09 | <0.1 |
| HCs | Total | Dou et al. 2021 | -0.803 | -0.967, -0.639 | <0.001 | 83.28 | 0.09 | <0.1 |
| HCs | Total | Echezerraga et al. 2017 | -0.814 | -0.97, -0.658 | <0.001 | 81.5 | 0.08 | <0.1 |
| HCs | Total | Hofer et al. 2017 | -0.769 | -0.93, -0.608 | <0.001 | 82.98 | 0.08 | <0.1 |
| HCs | Total | Lee et al. 2017 | -0.8 | -0.964, -0.636 | <0.001 | 83.69 | 0.09 | <0.1 |
| HCs | Total | Mizuno et al. 2016 | -0.8 | -0.964, -0.637 | <0.001 | 83.72 | 0.09 | <0.1 |
| HCs | Total | Park et al. 2023 | -0.783 | -0.952, -0.615 | <0.001 | 80.48 | 0.09 | <0.1 |
| HCs | Total | Post et al. 2018 | -0.769 | -0.93, -0.608 | <0.001 | 82.98 | 0.08 | <0.1 |
| HCs | Total | Tsigkaropoulou et al. 2023 | -0.761 | -0.917, -0.604 | <0.001 | 81.8 | 0.08 | <0.1 |
| HCs | Total | Uygun et al. 2020 | -0.789 | -0.953, -0.624 | <0.001 | 84.12 | 0.09 | <0.1 |
| HCs | Total | Vieira et al. 2020 | -0.777 | -0.943, -0.612 | <0.001 | 83.1 | 0.09 | <0.1 |
| HCs | Total | Sato et al. 2023 | -0.781 | -0.942, -0.619 | <0.001 | 83.85 | 0.09 | <0.1 |
| HCs | Total | Kang et al. 2024 | -0.816 | -0.973, -0.658 | <0.001 | 77.33 | 0.08 | <0.1 |
| MDD | Total | Aslan et al. 2005 | 0.046 | -0.231, 0.323 | 0.74 | 89.54 | 0.15 | <0.1 |
| MDD | Total | Chung et al. 2018 | 0.026 | -0.246, 0.299 | 0.85 | 88.22 | 0.14 | <0.1 |
| MDD | Total | Craba et al. 2023 | 0.044 | -0.233, 0.322 | 0.75 | 89.49 | 0.15 | <0.1 |
| MDD | Total | Favale et al. 2023 | 0.142 | -0.067, 0.35 | 0.18 | 81.6 | 0.07 | <0.1 |
| MDD | Total | Just et al., 2022 | 0.081 | -0.176, 0.338 | 0.54 | 88.95 | 0.13 | <0.1 |
| MDD | Total | Nunes et al. 2022 | 0.007 | -0.244, 0.258 | 0.96 | 86.16 | 0.11 | <0.1 |
| MDD | Total | Park et al. 2023 | 0.076 | -0.207, 0.359 | 0.6 | 86.69 | 0.15 | <0.1 |
| MDD | Total | Tsigkaropoulou et al. 2023 | 0.009 | -0.241, 0.258 | 0.95 | 86.69 | 0.11 | <0.1 |
| MDD | Total | Vieira et al. 2020 | 0.115 | -0.14, 0.369 | 0.38 | 85.99 | 0.12 | <0.1 |
| MDD | Total | Kang et al. 2024 | 0.068 | -0.217, 0.353 | 0.64 | 87.04 | 0.15 | <0.1 |
| SCZ | Total | Datta & Chetia. 2023 | 0.344 | 0.018, 0.67 | 0.039 | 69.78 | 0.08 | <0.1 |
| SCZ | Total | Deng et al. 2018 | 0.246 | 0, 0.492 | 0.05 | 42.12 | 0.03 | 0.17 |
| SCZ | Total | Hofer et al. 2017 | 0.438 | 0.251, 0.625 | <0.001 | 0 | 0 | 0.41 |
| SCZ | Total | Mizuno et al. 2016 | 0.303 | -0.032, 0.637 | 0.08 | 66.13 | 0.08 | <0.1 |
| SCZ | Total | Nunes et al. 2022 | 0.346 | -0.009, 0.701 | 0.06 | 66.87 | 0.09 | <0.1 |

## Main analysis, single-group meta-analysis

The forest plots for each meta-analysis are available at https://osf.io/qxh3y/?view_only=d0f7414073fe41c08b5f595a81ed33b4.

## Meta-regression analyses, single-group meta-analysis

**Table 6 - Meta-regression analyses**

The results are highlighted in green when a higher value of that specific predictor is significantly associated with a higher resilience score.

The results are highlighted in blue when a higher value of that specific predictor is significantly associated with a lower resilience score.

| **Outcome type** | **Predictor** | **Studies, n** | **Beta** | **95% CIs** | **p-value** | **Score at predictor, lower value** | **Score at predictor, higher value** |
| --- | --- | --- | --- | --- | --- | --- | --- |
| CD-RISC, Total | Percentage of females | 12 | -0.162 | -1.099, 0.775 | 0.74 | 56.012 at 36.7 | 50.524 at 70.6 |
| CD-RISC, Total | Mean age | 11 | -0.818 | -1.638, 0.003 | 0.05 | 67.91 at 22.71 | 32.786 at 65.67 |
| CD-RISC, Total | Duration of illness | 8 | -0.357 | -1.583, 0.869 | 0.57 | 52.62 at 7.56 | 41.697 at 38.13 |
| CD-RISC, Total | Percentage of people with BD1 | 8 | 0.218 | -0.24, 0.677 | 0.35 | 44.975 at 22.1 | 59.804 at 90 |
| CD-RISC, Total | MADRS symptoms severity | 5 | -2.087 | -3.496, -0.678 | 0.004 | 64.797 at 3.8 | 24.727 at 23 |
| CD-RISC, Total | YMRS symptoms severity | 5 | 2.118 | -2.475, 6.71 | 0.37 | 46.806 at 1.04 | 63.791 at 9.06 |
| CD-RISC, Total | Age at onset | 4 | 0.308 | -0.571, 1.187 | 0.49 | 57.304 at 21.22 | 60.066 at 30.2 |
| RS-25, Total | Mean age | 6 | 0.008 | -1.402, 1.418 | 0.99 | 124.075 at 25.78 | 124.28 at 50.2 |
| RS-25, Total | Percentage of females | 6 | -0.49 | -1.7, 0.719 | 0.43 | 129.495 at 53.3 | 118.855 at 75 |
| RS-25, Total | Duration of illness | 5 | 1.286 | -4.267, 6.839 | 0.65 | 120.248 at 8.4 | 129.765 at 15.8 |
| RS-25, Total | MADRS symptoms severity | 3 | 0.166 | -2.507, 2.838 | 0.9 | 123.278 at 4.6 | 124.505 at 12 |
| RS-25, Total | Percentage of people with BD1 | 3 | 0.268 | 0.166, 0.37 | <0.001 | 103 at 0 | 129.8 at 100 |
| RS-25, Total | YMRS symptoms severity | 3 | -23.327 | -55.803, 9.148 | 0.16 | 126.083 at 1.2 | 105.088 at 2.1 |
| RSA, Total | Percentage of females | 5 | -1.204 | -6.794, 4.386 | 0.67 | 105.206 at 43 | 72.694 at 70 |
| RSA, Total | Percentage of people in euthymia | 5 | 0.04 | -1.286, 1.366 | 0.95 | 87.3 at 0 | 91.293 at 100 |
| RSA, Total | Mean age | 4 | -3.28 | -6.392, -0.167 | 0.039 | 131.443 at 32.7 | 86.513 at 46.4 |
| RSA, Total | Percentage of people with BD1 | 4 | 0.648 | -0.187, 1.483 | 0.13 | 87.3 at 48.7 | 120.54 at 100 |
| RSA, Total | Duration of illness | 3 | -2.715 | -5.757, 0.328 | 0.08 | 140.284 at 1.9 | 96.034 at 18.2 |

## Sensitivity analyses, leave-one-out sensitivity analysis, single-group meta-analysis

**Table 7 - Leave-one-out sensitivity analysis**

| **Outcome type** | **Author, year of the study removed** | **Raw mean** | **95% CIs** | **I2** | **tau2** | **Q test p-value** |
| --- | --- | --- | --- | --- | --- | --- |
| CD-RISC, Total | Bozikas et al. 2018 | 51.329 | 41.923, 60.736 | 99.12 | 272.05 | <0.1 |
| CD-RISC, Total | Choi et al. 2015 | 51.452 | 42.005, 60.899 | 99.15 | 274.54 | <0.1 |
| CD-RISC, Total | Chung et al. 2018 | 51.66 | 42.157, 61.164 | 99.16 | 277.99 | <0.1 |
| CD-RISC, Total | Craba et al. 2023 | 51.932 | 42.374, 61.49 | 99.15 | 280.98 | <0.1 |
| CD-RISC, Total | Datta & Chetia. 2023 | 49.995 | 41.583, 58.407 | 98.93 | 216.95 | <0.1 |
| CD-RISC, Total | Deng et al. 2018 | 51.392 | 41.977, 60.807 | 99.16 | 272.98 | <0.1 |
| CD-RISC, Total | Dou et al. 2021 | 51.862 | 42.307, 61.417 | 99.11 | 280.63 | <0.1 |
| CD-RISC, Total | Favale et al. 2023 | 55.106 | 47.98, 62.232 | 98.36 | 154.06 | <0.1 |
| CD-RISC, Total | Lee et al. 2017 | 51.454 | 42.004, 60.904 | 99.14 | 274.64 | <0.1 |
| CD-RISC, Total | Park et al. 2023 | 52.7 | 43.189, 62.211 | 98.91 | 277.9 | <0.1 |
| CD-RISC, Total | Tsigkaropoulou et al. 2023 | 51.931 | 42.38, 61.483 | 99.17 | 280.79 | <0.1 |
| CD-RISC, Total | Montejo et al. 2024 | 54.353 | 46.019, 62.688 | 98.8 | 212.45 | <0.1 |
| CD-RISC, Total | Kang et al. 2024 | 52.7 | 43.19, 62.211 | 98.92 | 277.89 | <0.1 |
| RS-25, Total | Hofer et al. 2017 | 123.096 | 111.772, 134.42 | 94.95 | 156.74 | <0.1 |
| RS-25, Total | Mizuno et al. 2016 | 125.134 | 113.685, 136.582 | 94.28 | 159.56 | <0.1 |
| RS-25, Total | Nunes et al. 2022 | 121.367 | 112.414, 130.32 | 91.85 | 94.27 | <0.1 |
| RS-25, Total | Post et al. 2018 | 123.096 | 111.772, 134.42 | 94.95 | 156.74 | <0.1 |
| RS-25, Total | Vieira et al. 2020 | 124.599 | 112.982, 136.216 | 94.91 | 165.07 | <0.1 |
| RS-25, Total | Sato et al. 2023 | 128 | 121.32, 134.681 | 87.38 | 50.52 | <0.1 |
| RSA, Total | Kesebir et al. 2015 | 79.513 | 27.098, 131.928 | 99.97 | 2858.81 | <0.1 |
| RSA, Total | Palagini et al. 2022 | 91.293 | 31.994, 150.592 | 99.95 | 3658.75 | <0.1 |
| RSA, Total | Şenormanci et al. 2020 | 81.04 | 26.75, 135.331 | 99.97 | 3066.88 | <0.1 |
| RSA, Total | Uygun et al. 2020 | 88.39 | 29.308, 147.473 | 99.97 | 3632.72 | <0.1 |
| RSA, Total | Mackali et al. 2023 | 112.199 | 89.911, 134.487 | 99.5 | 514.26 | <0.1 |

# APPENDIX VII

## Publication bias, two-groups meta-analysis

The funnel plots for each meta-analysis are available at https://osf.io/qxh3y/?view_only=d0f7414073fe41c08b5f595a81ed33b4

**Table 7 - Publication bias**

| **Control group** | **Outcome type** | **Egger's z** | **p-value** |
| --- | --- | --- | --- |
| HCs | Total | -1.783 | 0.07 |
| MDD | Total | -0.799 | 0.42 |

## Publication bias, single-group meta-analysis

The funnel plots for each meta-analysis are available at https://osf.io/qxh3y/?view_only=d0f7414073fe41c08b5f595a81ed33b4

**Table 8 - Publication bias**

| **Outcome type** | **Egger's z** | **p-value** |
| --- | --- | --- |
| CD-RISC, Total | 2.893 | 0.004 |
